# Testing the Impact of Robotic Lawn Mowers on European Hedgehogs (*Erinaceus europaeus*) and Designing a Safety Test

**DOI:** 10.3390/ani14010122

**Published:** 2023-12-29

**Authors:** Sophie Lund Rasmussen, Bettina Thuland Schrøder, Anne Berger, Rahel Sollmann, David W. Macdonald, Cino Pertoldi, Aage Kristian Olsen Alstrup

**Affiliations:** 1 Wildlife Conservation Research Unit, The Recanati-Kaplan Centre, Department of Biology, University of Oxford, Tubney House, Abingdon Road, Tubney, Abingdon OX13 5QL, UK; david.macdonald@biology.ox.ac.uk; 2 Department of Chemistry and Bioscience, Aalborg University, Fredrik Bajers Vej 7H, 9220 Aalborg, Denmark; cp@bio.aau.dk; 3 Linacre College, University of Oxford, St. Cross Road, Oxford OX1 3JA, UK; 4 Behavioral Ecology Group, Section for Ecology and Evolution, Department of Biology, University of Copenhagen, Universitetsparken 15, 2100 Copenhagen Ø, Denmark; catsandconservation@gmail.com; 5 Department of Evolutionary Ecology, Leibniz Institute for Zoo and Wildlife Research, Alfred-Kowalke-Straße 17, 10315 Berlin, Germany; berger@izw-berlin.de; 6 Department of Ecological Dynamics, Leibniz Institute for Zoo and Wildlife Research, Alfred-Kowalke-Straße 17, 10315 Berlin, Germany; sollmann@izw-berlin.de; 7 Aalborg Zoo, Mølleparkvej 63, 9000 Aalborg, Denmark; 8 Department of Nuclear Medicine and PET, Aarhus University Hospital, Palle Juul-Jensens Boulevard 165, 8200 Aarhus, Denmark; aagealst@rm.dk; 9 Department of Clinical Medicine, Aarhus University, Palle Juul-Jensens Boulevard 165, 8200 Aarhus, Denmark

**Keywords:** European hedgehog, *Erinaceus europaeus*, robotic lawn mowers, wildlife conservation, safety tests, garden technology, anthropogenic disturbance, lawn care

## Abstract

**Simple Summary:**

The declining populations of European hedgehogs (*Erinaceus europaeus*) are increasingly inhabiting areas with human occupation. However, sharing habitats with humans comes at a cost: a residential garden holds many potential dangers for hedgehogs. Previous research has shown that certain models of robotic lawn mowers may harm hedgehogs. This study investigated the effects of 19 models of robotic lawn mowers on hedgehog cadavers. The insights gained from the current and previous research led to the design of a protocol for testing the safety of robotic lawn mowers on hedgehogs. The proposed standardised safety test will hopefully be implemented in the European Committee for Electrotechnical Standardization (CENELEC) protocol, potentially allowing for a labelling system indicating whether a robotic lawn mower is safe for hedgehogs, guiding the consumers to purchase hedgehog-friendly robotic lawn mowers in the future, thus reducing the negative impact some models of robotic lawn mowers may have on hedgehog conservation.

**Abstract:**

Previous research has established that some models of robotic lawn mowers are potentially harmful to hedgehogs. As the market for robotic lawn mowers is expanding rapidly and the populations of European hedgehogs (*Erinaceus europaeus*) are in decline, it is important to investigate this risk further to understand the potential threat which some robotic lawn mowers may pose to hedgehogs. We tested 19 models of robotic lawn mowers in collision with hedgehog cadavers to measure their effect on hedgehogs. Our results showed that some models of robotic lawn mowers may injure hedgehogs, whereas others are not harmful to them. Apart from one single incidence, all robotic lawn mowers had to physically touch the hedgehog carcasses to detect them. Larger hedgehog cadavers were less likely to be “injured”, with height being the most influential measure of size. The firmness of the tested hedgehog cadavers (frozen or thawed) did not influence the outcome of the collision tests. Neither the position of the hedgehog cadavers nor the selected technical features of the lawn mowers affected the probability of injury. Based on the results, we designed a standardised safety test to measure the effect of a specific model of robotic lawn mower on hedgehogs.

## 1. Introduction

The European hedgehog (*Erinaceus europaeus*) is in documented decline in several western European countries [1,2,3,4,5,6,7,8,9,10,11]. Previous research has unravelled a variety of suspected causes for the decline, such as road traffic accidents, habitat loss, habitat fragmentation, inbreeding, intensified agricultural practices, a reduction in biodiversity (and thereby natural food items), lack of suitable nest sites in residential gardens, accidents caused by garden tools, netting and other anthropogenic sources in residential gardens, infections with pathogens and endoparasites, badger predation, and finally, molluscicide and rodenticide poisoning [2,12,13,14,15,16,17,18,19,20,21,22,23,24,25,26,27,28,29,30,31,32]. These factors combined reduce the mean age of hedgehogs to two years (see Rasmussen et al. [33] Table 1 for an overview), despite their potential to reach up to 16 years of age [33]. To optimise the conservation initiatives directed at this species, there is a need for further investigation of the drivers behind this worrying decline in hedgehog populations.

### 1.1. Hedgehogs and Robotic Lawn Mowers

Research indicates that hedgehogs are nowadays increasingly associated with human occupation [7,17,18,34]. Unfortunately, sharing habitats with humans comes at a cost, as residential gardens provide many anthropogenic sources of danger to hedgehog survival. One of these potentially harmful features is certain models of robotic lawn mowers [26,35,36]. With robotic lawn mowers becoming increasingly popular throughout the distribution range of hedgehogs in Europe, there is a high likelihood for a hedgehog to encounter numerous robotic lawn mowers throughout its lifespan. The risk is heightened because some garden owners let the machines run after sunset, which is convenient for the human residents but coincides with the activity period of the nocturnal hedgehogs. Market insight reports predict that the global robotic lawn mower market will expand from USD 0.8–1.5 billion in 2020–2022 to USD 2.7–4 billion in 2032 with an anticipated compound annual growth rate (CAGR) of 11.5–15.5% during the forecast period [37,38]. This calls for an effort to eliminate any models of robotic lawn mowers which can potentially harm hedgehogs, to mitigate the negative effect these products may pose on hedgehog conservation. However, this endeavour requires research to inform the manufacturers in their development of more hedgehog-friendly robotic lawn mowers, alongside the design of a standardised safety test to evaluate and approve new models of robotic lawn mowers for the market, in terms of hedgehog safety, as an addition to the current mandatory general safety guidelines provided in the European Committee for Electrotechnical Standardization (CENELEC) protocol [39].

### 1.2. Study Aims

In response to the background information introduced in the previous sections, the aims of this study were as follows:

- Gain further insight on the effects on hedgehogs through collision tests of a selection of robotic lawn mowers available for purchase on the European market, representing different technical specifications, brands, and price ranges.
- Define any technical features in the robotic lawn mowers which may increase the safety for hedgehogs to guide the manufacturers in the design of more hedgehog-friendly machines.
- Obtain the necessary knowledge through the tests to design the optimal standardised safety test, such as the following:
  o The number of test replications needed to provide reliable results;
  o The ideal size and composition of a future hedgehog crash test dummy;
  o The optimal combination of test positions to represent the most realistic scenarios of encounters between hedgehogs and robotic lawn mowers.
- To propose a protocol for a standardised safety test to measure the effect of a specific model of robotic lawn mower on hedgehogs.

## 2. Materials and Methods

Prior to the tests, we contacted the different manufacturers of robotic lawn mowers offering to include their products in our experiments, but only two (STIHL and Husqvarna) provided robotic lawn mowers for the research. We tested a total of 19 models of robotic lawn mowers in this study. The selection was influenced by the availability of the products at the test facilities and was furthermore based on the advice of a product specialist in robotic lawn mowers. The mowers chosen for this study are considered to represent a broad spectrum of brands, models, and specifications of the products available on the European market (Table 1). We also prioritised the inclusion of as many as possible of the models tested by Rasmussen et al. (2021) [26] to facilitate comparisons between the different tests. The cutting heights of the machines were set to represent the standard settings recommended for each product and are described in Table 1.

Of the 19 robotic lawn mowers tested, 2 had fixed blades and 17 had pivoting blades (please see Figure 1A,B in Rasmussen et al. (2021) [26] for pictures of the types of blades).

The robotic lawn mower tests were performed on dead hedgehogs, henceforth referred to as “hedgehogs”. These animals had died in care, primarily due to infections, at hedgehog rehabilitation centres in Denmark from May to November 2022. All hedgehogs chosen for this study were intact with no visible injuries prior to the tests. The hedgehogs were stored in freezers at −20 °C and were thawed before the regular tests. The hedgehog cadavers all weighed between 250 and 600 g, representing the age group of recently independent juvenile hedgehogs, equivalent to the weight class 2 described by Rasmussen et al. (2021) [26] Table 2. This weight class was chosen as it yielded the most diverse results, with a larger variation between the different positions compared to individuals of other weight classes, in the tests performed by Rasmussen et al. (2021) [26].

**Figure 1:**
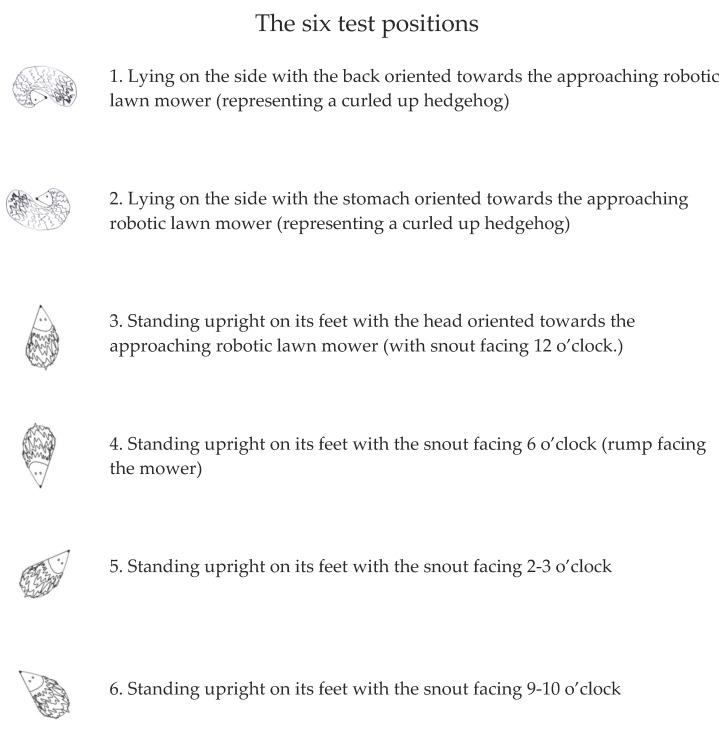
An overview of the six different test positions used during the tests. Only hedgehog cadavers were used in these tests.

Based on the results reported by Rasmussen et al. (2024) [36] testing the behaviour of live hedgehogs facing a disarmed, robotic lawn mower, each individual was tested in six different positions (Figure 1) in an attempt to mimic the behaviour of a live individual. The most commonly recorded position during the tests on live hedgehogs was “upright position with snout pointing inwards” (43%) [36] which could not be properly mimicked with a dead hedgehog as the head would not bend inwards and stay in place, leaving us to combine this with the second most frequently recorded behaviour (20%), test position 3:

The tests were recorded with two Ring Stick Up Cam^®^ (Ring^TM^, Santa Monica, CA, USA) cameras placed on tripods.

Each model of robotic lawn mower was tested on one hedgehog. If an individual was injured by the mower during a test, the injuries were documented with the cameras. In most cases, the individual would thereafter be discarded to avoid the misinterpretation of previously sustained injuries in the subsequent tests.

The tests of 17 out of 19 machines were carried out in a test hall at Husqvarna headquarters in Huskvarna, Jönköping, Sweden, from 23 to 25 March 2023. The remaining two models (STIHL iMOW 7 and STIHL iMOW 5 (STIHL, Stuttgart, Germany)) were tested in a private garden in Lejre, Denmark, on 10 October 2023, as they could not be made available for the tests taking place in March 2023. All tests were performed during daylight hours.

**Figure 2:**
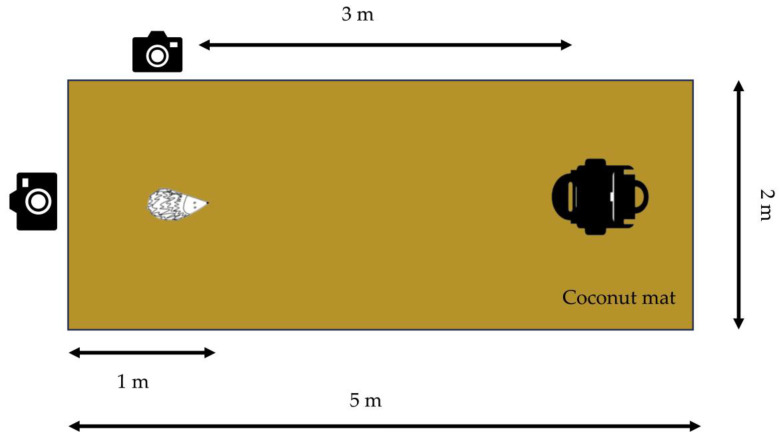
An overview of the setup for the test scenario. Only dead hedgehogs were used in the tests.

The tests were performed on a firm base of either concrete flooring, garden tiles, (STIHL iMOW 7 and STIHL iMOW 5) or asphalt (Segway NaviMow H3000E (Seqway Inc., Beijing, China) and Stiga Stig-A 1500 (Stiga, Castelfranco Veneto, Italy)), on a coconut mat with a rubber-backed base (dimensions 2 m in width and 5 m in length and 20 mm in height [40]). The coconut mat is the recommended base for the robotic lawn mower safety tests described in the European Committee for Electrotechnical Standardization (CENELEC) protocol [39]. The hedgehog was placed on the coconut mat lying 1 m from the edge of the mat and at a 3 m distance from the robotic lawn mower (Figure 2). The cameras were placed next to the hedgehog on the left-hand side at a 1 m distance from the hedgehog and behind it at a distance of 1 m. The mower was then turned on and manually directed to move towards the hedgehog. The distance of 3 m was sufficient to ensure the machine was operating at maximum speed and the blades were in action before reaching the hedgehog. If the machine did not move in a straight line towards the hedgehog, it was then relocated back to the initial position and turned on again. This was conducted to standardise the tests and to ensure that the hedgehog was located at the centre of the front of each approaching machine. In order to test certain models, the distance between the robotic lawn mower and the hedgehog deviated from the standard 3 m as a longer distance was required before the knives started rotating (3.4 m: Husqvarna Automower ^®^ Nera, Husqvarna Automower ^®^ Aspire R4 (Husqvarna, Huskvarna, Sweden)) or there was a need for a shorter distance to ensure the mower approached the hedgehog at the right angle (2 m: Husqvarna Automower ^®^ 105, Husqvarna Automower ^®^ 305 (Husqvarna, Huskvarna, Sweden), Gardena Sileno Life (Gardena GMBH, Ulm, Germany)).

In the cases of two models using satellite navigation (tests on Segway NaviMow H3000E and Stiga Stig-A 1500), the tests were performed outdoors with an asphalt concrete base below the coconut mat. In some instances, the machines were switched on at another distance than the standard 3 m from the hedgehog cadaver (Segway NaviMow H3000E: all tests at a 5 m distance). This was necessary because this particular model of robotic lawn mower require longer distances to gain momentum for the blades to be rotating at full speed (Segway NaviMow H3000E). For the tests of the Stiga Stig-A 1500 model, the movement algorithm of the machine was unpredictable, forcing the research team to manually place the coconut mat and hedgehog in front of the approaching lawn mower. In all tests, the Stiga Stig-A 1500 was fully up and running when the hedgehog and coconut mat were placed in front of it, at either a 2 m distance (test position 1–5) or a 4 m distance (test position 6).

### 2.1. Quantifying the Damage

We described the results of the tests, quantifying the severity of damage caused by the robotic lawn mowers, by allocating each outcome to one of five damage categories (Table 2):

### 2.2. Additional Comparison Tests

It was decided to add two types of comparison tests to the testing procedure. During early tests, it appeared that the size of the hedgehog carcasses used could potentially influence the results, where the smaller ones (<400 g in weight) would more frequently be injured compared to individuals of a larger size (>400 g). Therefore, we decided to perform additional comparison tests on larger hedgehog carcasses for the models of robotic lawn mowers which were previously tested on smaller hedgehogs (<400 g). Due to the limited number of individuals available for the comparison test, we most often only performed the test in position 3 to increase the likelihood of having intact carcasses available for tests on several models of robotic lawn mowers.

To investigate whether the firmness of the carcass would influence the results, we added a test on a frozen hedgehog carcass in test position 3 to the testing procedure for the machines chosen for comparison tests. For the tests performed in Lejre, Denmark, no frozen hedgehogs were available for testing, so this test type was omitted (STIHL iMOW 7 and STIHL iMOW 5).

### 2.3. Data Analyses

For our analyses, we combined test data from 2020 (published in Rasmussen et al. (2021) [26]) and the results produced in the present experiment in 2023. In contrast to the current experiments, the tests performed in 2020 only used three of the six positions and only tested on thawed hedgehog carcasses. Because of the limited amount of data for each lawn mower model and the categorical nature of the response variable (damage category 0–4), we transformed the response variable y to binary, with y = 1 when a test resulted in damage category 4 (i.e., hedgehog sustained injury in collision with the lawn mower, see definition in Table 2) and y = 0 otherwise (damage category 0–3). We analysed these data with a logistic regression, thus estimating the probability that a test resulted in injury, and the effects of several predictor variables on that probability.

The raw data suggested differences in injury probability among lawn mower models and we used ‘lawn mower model’ as a random intercept in all models; we refer to the model with the random intercept only as the base model. To evaluate the importance of a predictor for injury probability, we compared models with a predictor to the base model using Akaike’s Information Criterion (AIC; [41]). We further gauged the effect strength based on the coefficient estimate and evidence for the effect based on its *p*-value.

All models were fit in R v. 4.2.1 [42] using the lme4 package v. 1.1.30 [43].

#### 2.3.1. Additional Comparison Tests

We compared the outcomes of the tests on frozen hedgehog carcasses to unfrozen hedgehog carcasses. Due to the limited sample size of frozen hedgehogs (*n* = 12), we fit a separate logistic regression comparing injury probability between thawed and frozen hedgehogs, accounting only for lawn mower model, but none of the other predictor variables were found to affect injury probability (see Results). We excluded frozen hedgehogs from our main analysis investigating the effect of predictor variables on injury probability (see Section 2.3.2, Section 2.3.3, Section 2.3.4 and Section 2.3.5). We combined data from the comparison tests using thawed hedgehogs of different sizes (375–419 g) with data from the main tests for our main analysis.

#### 2.3.2. Investigating Potential Differences in Injury Probability Depending on the Position of the Hedgehog

During the tests, the hedgehogs were placed in six positions relative to the direction of approach of the robotic lawn mower (see Figure 1 for a description of the six positions). To test whether there were differences in the probability of injury depending on the position of the hedgehog, we prepared the following model. To reduce the number of levels of this categorical predictor, we grouped positions into 3 categories: 1 + 2 (lying on the side), 3 + 4 (standing, in line with mower), and 5 + 6 (standing, at angle with mower). We included this new position variable as a predictor of injury category.

#### 2.3.3. Comparing the Results of the 2020 and 2023 Tests

Some of the models of robotic lawn mowers were tested in 2020 [26] as well as in the current experiment. The test scenarios did differ slightly between years, as the hedgehog carcasses used in the different tests were not identical, and the tests were performed in different locations with different ground covers (lawns in 2020 and coconut mats in 2023). Therefore, to test whether there were differences in the probability of injury between the 2020 and 2023 tests, we added a categorical year effect to the base model. Because positions 4–6 were not used in tests in 2020 and there was some (albeit weak) evidence that positions 5 + 6 may have a different injury probability (see Results), we also included ‘position’ in this model. This was to avoid any confounding effect between ‘year’ and ‘position’.

#### 2.3.4. Measuring the Effect of the Size of Hedgehog Carcass Used in the Tests on the Probability of Sustaining Injury

Even though the hedgehog carcasses used for the tests all matched the weight category 250–600 g representing independent juvenile hedgehogs, there was still a large variation in size between them. This led us to test whether the characteristics of the hedgehog carcass affect its probability to sustain injury during the tests. Three measures were collected for the hedgehogs included in the tests: weight (g), height (cm), and circumference (cm). The measurements in cm were recorded as the maximum height and the maximum circumference of the hedgehog. The latter two measures were not collected for any of the hedgehogs tested in 2020. The three measures of size were strongly correlated. To assess via AIC which of the size predictors were the most important, we subset the data to include only those trials where all three measures were taken (*n* = 147 tests). To test whether there were thresholds or optimum relationships between the probability of injury and hedgehog characteristics, we fit models with linear and quadratic effects separately for each predictor (weight, height, and circumference). We compared the six models with linear or quadratic predictors to the base model. We centred and scaled all measures prior to analysis.

#### 2.3.5. Testing Whether the Technical Features of the Robotic Lawn Mowers Affect the Probability of Causing Injury to Hedgehogs

A range of different technical features were registered for the robotic lawn mowers included in the tests (see Table 1). In our analysis to test for effects of these features on the probability of causing injury to hedgehogs, we excluded ‘camera vision’, as none of the robotic lawn mowers tested had it. We included cutting height as a continuous predictor and all other attributes as categorical (binary) predictors. Because there was very little variation in hedgehog characteristics for each robotic lawn mower (each year, a mower was typically tested only with a single hedgehog), and we wanted to avoid confounding effects of hedgehog characteristics and lawn mower attributes, we included the most important hedgehog characteristic from 2.3.4 (height) in all lawn mower attribute models. To do so, we subset the data to those tests which included records of hedgehog characteristics (*n* = 147); all lawn mower attributes were always recorded for these tests.

#### 2.3.6. Calculating the Optimal Number of Tests to Characterise the Risk of Injury to Hedgehogs Caused by a Specific Robotic Lawn Mower

Measuring categorical data (damage categories), no single damage category alone would characterise a lawn mower model. Rather, each model would have a set of probabilities of how likely each damage category is to occur in a trial. With the current test setup, this would be a set of five probabilities (five damage categories) for each mower, therefore requiring much more data to estimate these probabilities precisely. To reduce this challenge, we again limited the damage categories to injured (category 4) and not injured (category 0–3), focusing on estimating the probability of a lawn mower model causing injury.

One of our goals was to characterise each lawn mower model based on the risk it poses to hedgehogs. Ideally, to do so, we would have included ‘lawn mower model’ as a fixed effect in the previously described analyses. However, for some models, no tests resulted in injuries (i.e., all y = 0), precluding estimating model-specific injury probabilities for these models with fixed effects. More importantly, overall, the data per model were sparse, which leads to uncertain estimates of model-specific injury probabilities. Therefore, for the application in future standardised safety tests, we wanted to apply our test results to determine the optimal number of test repeats (henceforth, sample size) necessary to confidently characterise each robotic lawn mower model’s risk of causing injury to hedgehogs.

To define the amount of data (trials per mower) needed to estimate the probability of injury precisely, a simulation-based approach, with the following steps, was used:

(a) We set input injury probabilities for all robotic lawn mower models based on the estimates from a logistic regression with the fixed effect of ‘lawn mower model’. We excluded those lawn mower models for which the regression could not estimate a model-specific injury probability.
(b) We created input values for the effects of hedgehog height (the most important characteristic to affect injury probability—see Results) and position on injury probability, using the results from the previously described analyses. Even though the effect of position on injury probability was weak (see Results), we chose to include it in our data simulation to mimic reality, as robotic lawn mowers may encounter hedgehogs in different positions.
(c) We used these input values to simulate new synthetic trial data for different sample sizes per robotic lawn mower model. In the original data, approximately 10 tests were performed per model, depending on whether the model was tested in a comparison test and how many positions were used in that particular test. In the simulations, we explored sample sizes of 25, 50, 75, 100, and 150 per robotic lawn mower model. For each trial, hedgehog height was randomly sampled from all unique heights represented in the dataset from the collision tests (eight different sizes); similarly, position was randomly sampled from the three grouped positions (1 + 2, 3 + 4, and 5 + 6, see Figure 1 for a description of the positions). For each sample size, we created 250 synthetic datasets.
(d) We analysed the synthetic data to estimate the specific injury probability for each model of robotic lawn mower. Specifically, we fit a logistic regression model with a fixed effect of ‘lawn mower model’, accounting for hedgehog height and height squared. The regression did not account for ‘position’, as position introduces realistic variability into the synthetic data, and the model estimates the average injury probability across all positions.
(e) We summarised the results across all 250 simulated datasets for each sample size scenario. Specifically, for each dataset, we determined estimated injury probability for each model of robotic lawn mower, which due to scaling of the height variable corresponds to the expected injury probability for an average-sized hedgehog. We calculated 95% confidence intervals (CIs) and the mean CI across all 250 datasets. We plotted average CI width against sample size to visualise how the level of uncertainty declines with increasing sample size.

## 3. Results

**Figure 3:**
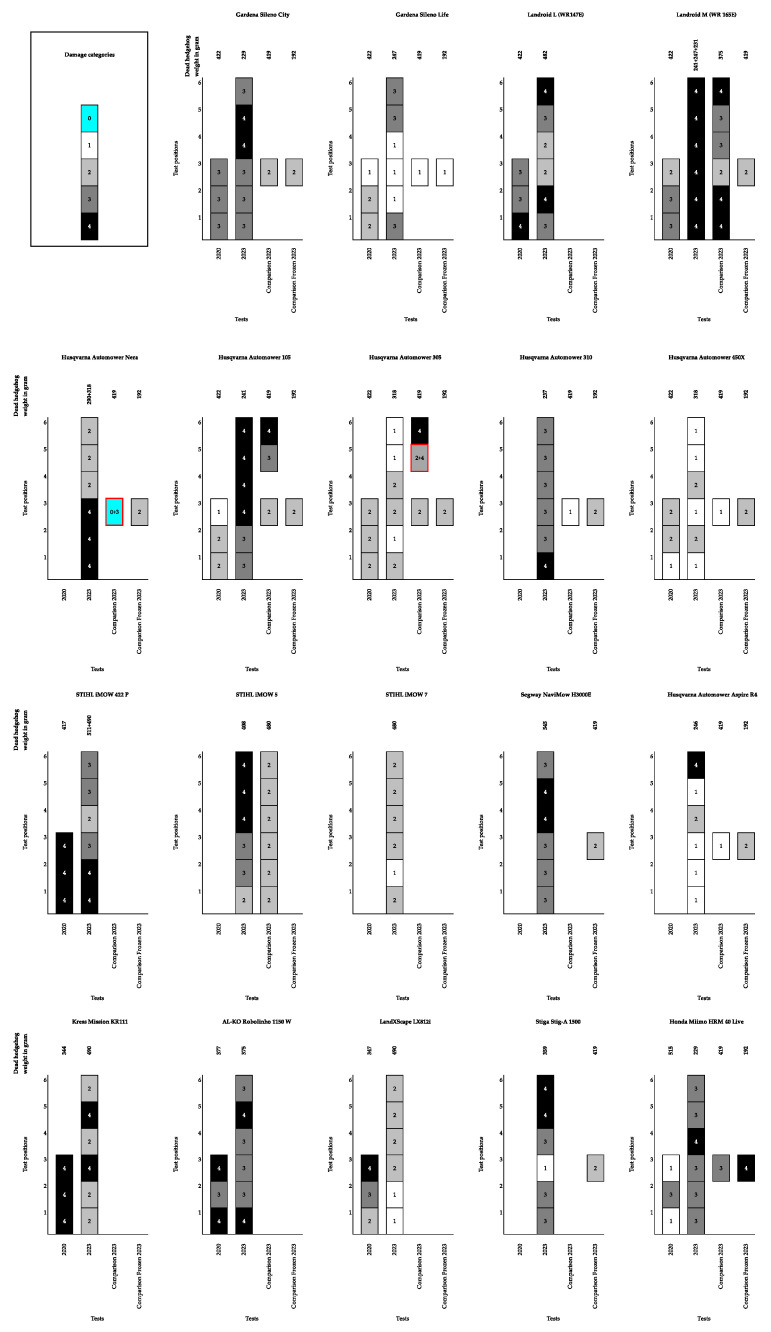
An overview of the test results for each of the 19 tested robotic lawn mowers. The results from the tests performed in 2020 are also visualised for the machines tested by Rasmussen et al. (2021) [26]. The *x*-axis illustrates the test categories (2020, 2023, comparison, and comparison with a frozen hedgehog), and the *y*-axis shows the six different test positions. All hedgehog carcasses used in the tests weighed between 250–600 g. The numbers within the fields of the columns denote the damage categories registered for each test position. The numbers above the columns describe the weight in g of the hedgehog carcass used for the specific test. A red highlight marking of the result box (Husqvarna Automower Nera and Husqvarna Automower 305) indicates that this test position and scenario was tested twice and yielded two different results, with colour describing the lowest of the measured damage categories presented in the box.

The results from the collision tests between the hedgehog carcasses and the 19 different models of robotic lawn mowers tested can be found in Figure 3. For comparison, the figure includes the results from the tests performed previously on the same weight category size of hedgehog carcasses in 2020 [26]. The results show that some of the robotic lawn mowers did cause injury to the hedgehog carcasses tested (damage category 4), whereas other models of robotic lawn mowers would push the hedgehog prior to detecting it, causing the robotic lawn mower to change direction without harming the hedgehog (damage categories 1–2). There was only one incidence of a damage category 0, where the hedgehog was apparently detected at a distance: the robotic lawn mower changed directions and did not come into contact with the hedgehog. However, the same result could not be replicated; when the test was repeated, it yielded a damage category 3. The full dataset is available in Table S1.

### 3.1. Additional Comparison Tests

The model comparing injury probability between frozen and thawed hedgehogs provided little evidence for an effect of the state of the hedgehog. The base model had essentially equal support as the model including the hedgehog state as a predictor (ΔAIC = 0.23); the coefficient estimate had a high uncertainty (1.396, SE = 1.07), and correspondingly, the *p*-value suggested that evidence in favour of this effect was weak (*p* = 0.192). We caution, however, that this may be a function of the low sample size of frozen hedgehogs and the resulting inability to account for important sources of variation in injury probability (see following sections) in this comparison.

### 3.2. Investigating Potential Differences in Injury Probability Depending on the Position of the Hedgehog

Evidence that hedgehog position affected injury probability was weak to nonexistent. Both the ‘position’ model and the base model had essentially equal support (ΔAIC = 0.5). The effect estimates for positions 3 + 4 had a large standard error, and both coefficients had non-significant *p*-values (Table 3). However, the effect of positions 5 + 6 appears stronger and more certain than that of positions 3 + 4.

### 3.3. Comparing the Results in the 2020 and 2023 Tests

There was no evidence that year affected the injury probability. The model with year had a higher AIC value than the base model (ΔAIC = 1.23), and the coefficient for tests being conducted in 2023 had a large standard error (beta = −0.26, SE = 0.49) and *p*-value (0.60).

### 3.4. Measuring the Effect of the Size of Hedgehog Carcass Used in the Tests on the Probability of Sustaining Injury

**Figure 4:**
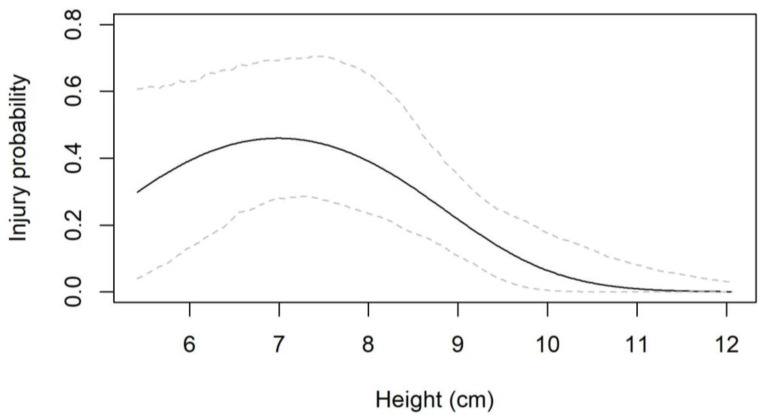
Relationship between hedgehog height and probability of being injured by a lawn mower. Dashed lines show confidence intervals.

The AIC values showed that out of the three characteristics, height was the most important predictor of injury probability; the model including a quadratic effect of height was considerably better than the one with the linear effect (Table 4). All other models (of weight and circumference) were similar or worse in AIC than the base model. Coefficient estimates from the quadratic height model showed that injury probability initially increased with height but then declined after about 7 cm of height (Figure 4).

### 3.5. Testing Whether the Technical Features of the Robotic Lawn Mowers Affect the Probability of Causing Injury to Hedgehogs

The only model whose AIC was lower than that of the base model was the one containing an effect of front- or rear-wheel drive, but the ΔAIC was 0.6, thus suggesting that this attribute did not improve the model (Table 5). The model indicated that rear-wheel drive caused higher injury probability, but the standard error was large (1.27, SE = 0.83), and the effect was non-significant (*p* = 0.13).

### 3.6. Determining the Optimal Number of Tests to Characterise the Risk of Injury to Hedgehogs Caused by a Specific Robotic Lawn Mower

**Figure 5:**
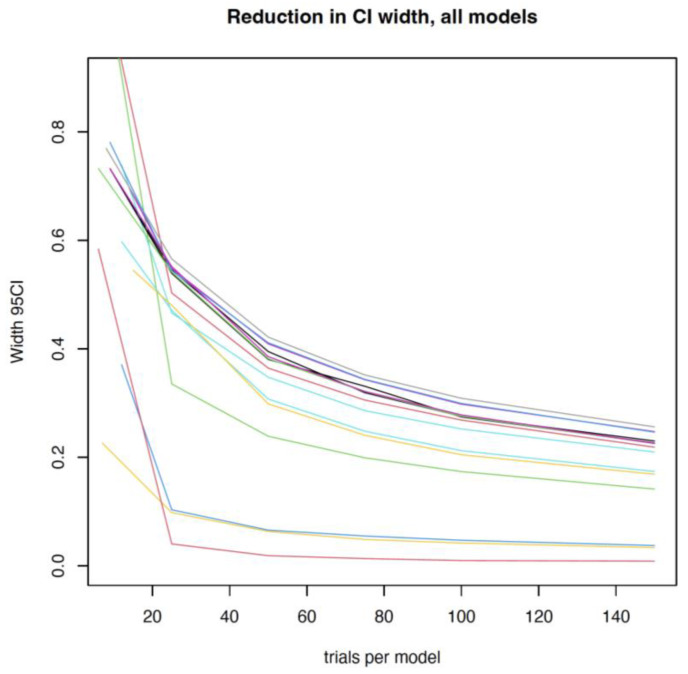
A visualisation of the reduction in confidence interval width (indicating a better representation) as a function of an increased sample size (number of tests) for all 15 robotic lawn mower models included in the analysis. Each mower is represented by a specific colour.

Out of the 19 lawn mower models, it was possible to estimate a mower-specific injury probability (as input value for the simulation) for 15. For the remaining four models (Gardena Sileno Life, Husqvarna Automower 450X, LandXScape LX812i, and STIHL iMOW 7), no (or extremely few) trials resulted in injury, rendering the model unable to estimate injury probabilities for these mowers, causing an exclusion of these models from the simulation. As expected, for the remaining models of robotic lawn mowers, the level of uncertainty around their injury probability declined as the sample size (trials per model) was increased (Figure 5). However, that decline depended on the injury probability: the decline was less pronounced in models with very high or very low injury probability. Generally, the gains in certainty declined considerably after sample sizes of 50, causing us to suggest a test number of 50 to confidently characterise the risk of injury to hedgehogs caused by a specific lawn mower.

### 3.7. Using the Results to Design a Standardised Safety Test

#### 3.7.1. Size of the Hedgehog Crash Test Dummies

Based on the test results, indicating that the size of the hedgehog affects the outcome of a collision test, we suggest that a future standardised safety test measuring the effect of a specific model of robotic lawn mower on hedgehogs would include two sizes of hedgehog crash test dummies: one representing an independent juvenile hedgehog <400 g and 7 cm in height and another representing an adult hedgehog >600 g and ≥10 cm in height.

#### 3.7.2. Positions Used in the Tests

The findings of Rasmussen et al. (2024) [36] showed that live hedgehogs tend to either run away or position themselves in positions 3, 5, and 6 (Figure 1) when approached by a robotic lawn mower. As our tests showed that the position did not significantly influence the outcome of the collision tests, and as position 1 and 2 (curled up hedgehogs) would be challenging to mimic with a non-flexible hedgehog crash test dummy, we suggest excluding these two positions from the standardised safety test. As the hedgehog crash test dummy currently being prepared is designed without the features of a head, having completely similar front and back design, the position 4 could be considered redundant if the features of the front and back of the model are identical, as position 3 would therefore already have represented that position. As our results indicated a tendency for a higher probability of injury in positions 5 and 6, we recommend including both of these positions in a standardised safety test. Accordingly, we suggest using three positions for future standardised safety tests, namely positions 3, 5, and 6 (described in Figure 1).

#### 3.7.3. The Test Setup

Even though our results showed consistency between the 2020 and 2023 tests, performed on different surfaces (grass compared to a coconut mat placed on top of either a solid base of concrete, asphalt, or garden tiles), we recommend the test setup described in Figure 2. This furthermore serves to standardise the design, as lawns may differ in softness and grass height and plant composition. The proposed coconut mat is already a recommended standard base for the tests described in the CENELEC protocol [39].

#### 3.7.4. Number of Tests

As described previously, the optimal number of tests to characterise the risk of injury to hedgehogs caused by a specific robotic lawn mower is 50 or above. Therefore, we suggest that the standardised safety test should consist of 60 trials per size hedgehog crash test dummy to accommodate the three test positions chosen, testing each position 20 times.

#### 3.7.5. The Proposed Standardised Safety Test

**Figure 6:**
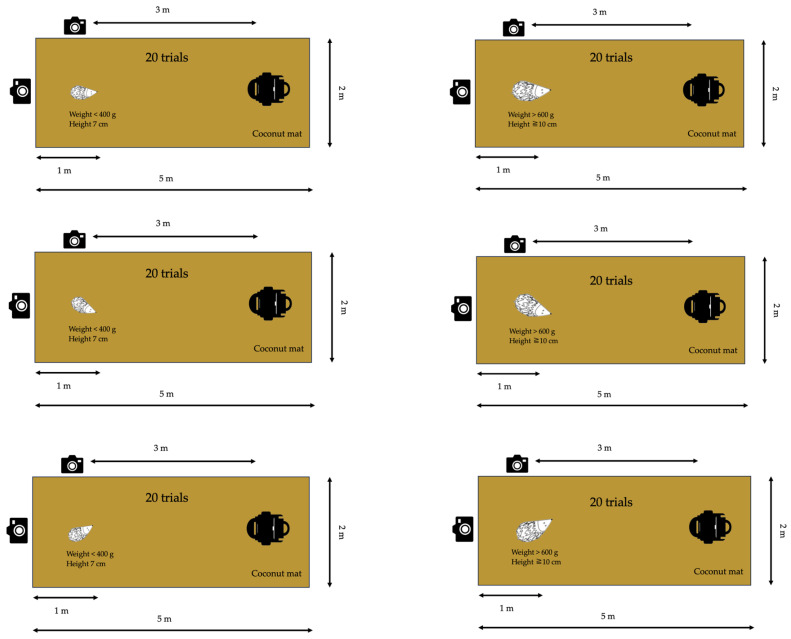
The suggested framework for a standardised safety test to measure the effect of a specific model of robotic lawn mower on hedgehogs.

Our suggestion is that the framework of a standardised safety test to measure the effect of a specific model of robotic lawn mower on hedgehogs is as follows (Figure 6):

- The tests shall be performed on concrete flooring on a coconut mat with a rubber-backed base (dimensions 2 m in width, 5 m in length, and 20 mm in height).
- The hedgehog crash test dummy shall be placed on the coconut mat lying 1 m from the edge of the mat and at a 3 m distance from the robotic lawn mower.
- Two cameras shall be positioned next to the hedgehog crash test dummy on the left-hand side at a 1 m distance from the dummy and behind the dummy at a distance of 1 m.
- Two sizes of hedgehog crash test dummies shall be used: <400 g and 7 cm in height and >600 g and ≥10 cm in height.
- Each hedgehog crash test dummy shall be tested in 60 trials:
  o 20 trials: Standing upright on its feet with the head oriented towards the approaching robotic lawn mower with the snout facing 12 o’clock;
  o 20 trials: Standing upright on its feet with the snout facing 2–3 o’clock;
  o 20 trials: Standing upright on its feet with the snout facing 9–10 o’clock.
- The interpretation of the results should be conducted as follows:
  o Robotic lawn mowers yielding only damage categories 0–2 in the tests (see Table 2 for a description of damage categories) should be labelled as safe for hedgehogs;
  o Models of robotic lawn mowers showing any results belonging to damage categories 3 and 4 cannot be labelled as safe for hedgehogs;
  o A robotic lawn mower fails the safety test if any of the results are classified as damage category 4.

## 4. Discussion

Our results showed that some of the robotic lawn mowers tested may injure hedgehogs, whereas others gave no evidence of being harmful to hedgehogs. Apart from one incidence, where we recorded damage category 0, all robotic lawn mowers had to physically interact with the hedgehog carcasses to detect them, whereafter some robotic lawn mowers changed direction and did not cause injury to the hedgehogs. Larger-sized hedgehogs were less likely to be injured, with height being the measure of size most useful in predicting injury. The firmness of the hedgehog cadavers (thawed or frozen) did not affect the outcome of the collision tests. There were no differences in test outcomes between the years 2020 and 2023, showing consistency in the results produced. There was little evidence that hedgehog position influenced injury probability and even less evidence that any of the selected technical features of the lawn mowers tested affected the probability of injury.

### 4.1. Hedgehog Crash Test Dummies as Alternatives to Hedgehog Carcasses in Future Tests

We found no difference between the outcomes of tests on frozen compared to thawed hedgehog carcasses, although keeping in mind that this comparison was based on a limited sample size. We decided to include the comparison test of frozen carcasses, as we considered a potential bias in the results which could arise if the hedgehog carcasses were softer than live hedgehogs due to the latter curling up and thereby tightening the muscles during a confrontation with a robotic lawn mower.

Work is currently underway to design an optimal hedgehog crash test dummy, mimicking a real, live hedgehog, to be used in the test setup. The dummy will be offered as a standard model for testing the safety of a robotic lawn mower on hedgehogs in the future. The ultimate goal is to provide an open-access recipe allowing relevant stakeholders, such as manufacturers of robotic lawn mowers and test institutes, to 3D print the crash test dummy and use this in the development of hedgehog-friendly robotic lawn mowers and for the standardised safety test we propose.

It is important to ensure that the hedgehog crash test dummy offered is realistic. The results of the present tests should inform the process of designing the dummy. In the worst-case scenario, a faulty dummy could cause misguided adjustments to the robotic lawn mower designs to the detriment of the hedgehogs. The degree of firmness of the hedgehog cadaver did not significantly affect the outcomes of the collision tests (frozen or thawed), encouraging us to conclude that a crash test dummy could be representative even though its composition is not identical to a real hedgehog. However, our results showed that the size of the hedgehog carcass does influence the risk of injury, which leads us to suggest that the hedgehog crash test dummy should be produced in two size categories: (1) <400 g and 7 cm in height (mimicking an independent juvenile hedgehog) and (2) >600 g and ≥10 cm in height (mimicking an adult hedgehog). Further work on the crash test dummy currently being developed is needed to ensure that the injury prediction model from the tests on dead hedgehogs applies directly to the dummy.

### 4.2. A Standardised Safety Test to Measure the Effect of a Specific Model of Robotic Lawn Mower on Hedgehogs

The goal is to have the standardised hedgehog safety test implemented in the CENELEC protocol [39] as a test offered for all robotic lawn mowers being approved for sale on the European market. The intention is to use this standardised safety test to establish an official labelling system for hedgehog-safe robotic lawn mowers, guiding the consumers to make the hedgehog-friendly choice when purchasing robotic lawn mowers.

One of the purposes of the present study was to compile sufficient data to provide a solid suggestion for a protocol for a standardised safety test to measure the effect of a specific model of robotic lawn mower on hedgehogs. Based on the information gathered in the present study, we have described our suggestion for a standardised safety test. This protocol should now be tested and validated, before being implemented in the CENELEC protocol (International Electrotechnical Commission (IEC), Technical Committee (TC) 116, Working Group (WG) 10, IEC 62841-4-X: Particular requirements for robotic lawnmowers).

### 4.3. The Safety of Robotic Lawn Mowers for Hedgehogs

Three years after the first tests on the effect of robotic lawn mowers on hedgehogs [26] and an increased focus on improving the safety of the robotic lawn mowers for hedgehogs in new models designed, we were pleased to see how the new models, tested for the first time, in general involved less harmful encounters with the hedgehog carcasses in our experiments. The damage category 0, where the robotic lawn mower detects the hedgehog at a distance and changes direction without coming into physical contact with it, should be regarded as the desired outcome. We did observe the damage category 0 in a single test. However, this result could not be replicated by repeating the test, which causes us to suggest that the robotic lawn mower may have detected (and avoided) a larger obstacle by chance, such as the camera recording the event, in the background. Regardless, the general reduction in harmful outcomes for the hedgehogs in the collision tests with new models and designs of robotic lawn mowers gives cause for optimism about the future. For now, our advice remains to restrict the running of robotic lawn mowers to daylight hours and check the lawn for any wildlife species which may be vulnerable in the encounter with a robotic lawn mower before turning on the machine.

## 5. Conclusions

Based on the experiments presented in this study, we conclude that some models of robotic lawn mowers may injure hedgehogs, whereas others are not harmful to them. Apart from one single incidence, all robotic lawn mowers had to physically touch the hedgehog carcasses to detect them. Height affected the risk of injury, with larger hedgehog cadavers being less likely to be damaged. The firmness of the tested hedgehog cadavers (frozen or thawed) did not influence the outcome of the collision tests. Neither the position of the hedgehog cadavers nor the selected technical features of the lawn mowers affected the probability of injury. The level of uncertainty regarding injury probability declined as the number of trials per model of robotic lawn mower was increased to a level of 50 tests, causing us to suggest a test number of 50 to characterise confidently the risk of injury to hedgehogs caused by a specific lawn mower. 

The insights provided by our results have enabled the design of a protocol for a standardised hedgehog safety test to quantify the effect of a given robotic lawn mower on hedgehogs. Used in combination with specially designed hedgehog crash test dummies, this protocol will hopefully lead to the development of more hedgehog-friendly robotic lawn mowers, thereby reducing the negative impact on hedgehogs.

## Figures and Tables

**Table 1:** Overview of features of the models of robotic lawn mowers tested and their cutting height settings. In the column “Blades”, P indicates “low energy pivoting blades” and F indicates “heavy duty fixed blades”.

Brand	Model	Blades	Collision Sensor	Wheel Motor Current Collision Detection	Wheels	Front (F)-/Rear (R)-Wheel Drive	Skid Plate	Headlights	Ultrasonic Sensors	Camera Vision	Cutting Height (cm)
AL-KO	1150	F	No	Yes	4	R	No	No	No	No	50
Gardena	Sileno City	P	No	Yes	3	F	No	No	No	No	58
Gardena	Sileno Life	P	No	Yes	4	F	No	No	No	No	35
Honda	HRM 40 Live	P	No	Yes	4	R	No	No	No	No	47
Husqvarna Automower ^®^	105	P	Yes	No	3	F	Yes	No	No	No	45
Husqvarna Automower ^®^	305 (310)	P	No	Yes	4	R	Yes	No	No	No	52
Husqvarna Automower ^®^	450X	P	Yes	No	4	R	Yes	Yes	Yes	No	60
Husqvarna Automower ^®^	310	P	Yes	No	4	R	Yes	No	No	No	65
Husqvarna Automower ^®^	Nera	P	Yes	No	4	R	Yes	Yes	Yes (Radar)	No	43
Husqvarna Automower ^®^	Aspire R4	P	No	Yes	3	F	No	No	No	No	50
Kress	KR111	P	Yes	No	4	R	No	No	Yes	No	45
LandXcape	LX812i	P	No	Yes	3	R	No	No	Yes	No	40
Segway NaviMow	H3000E	P	Yes	No	4	R	No	No	No	No	67
Stiga Stig-A	1500	P	Yes	No	4	R	No	No	No	No	35
Worx	Landroid L (WR153E)	P	No	Yes	4	R	No	No	No	No	60
Worx	Landroid M (WR143E)	P	No	Yes	4	R	No	No	Yes	No	60
STIHL	iMOW 422P	F	Yes	No	4	R	No	No	No	No	43
STIHL	iMOW 5	P	Yes	No	4	R	No	Yes	Yes	No	40
STIHL	iMOW 7	P	Yes	No	4	R	No	Yes	Yes	No	40

**Table 2:** A description of the five different damage categories used to describe the outcome of the different tests.

Damage Category	Description
0	No physical contact between the machine and the hedgehog. The machine senses the hedgehog from a distance, changes direction, and drives on without touching the hedgehog. No damage is caused to the hedgehog cadaver.
1	The robotic lawn mower approaches the hedgehog, and the front of the machine touches the hedgehog lightly (a “nudge”) and thereby detects the corpse. Immediately, the machine changes direction and drives on without touching the hedgehog further. No damage is caused to the hedgehog cadaver.
2	The robotic lawn mower approaches the hedgehog, and the front of the machine touches the hedgehog (a “flip”) to detect the hedgehog. The physical interaction causes the hedgehog to be moved into a different body position (flipped from lying on one side of the body to the other side of the body) or be lifted partly from the ground before settling in the same position again. Afterwards, the machine changes direction and drives on without touching the hedgehog further. The damage to the hedgehog is at most minimal and involves no contact with the blades (at worst this might cause a slight bruise).
3	The robotic lawn mower fails to detect the presence of the hedgehog and continues to drive across the hedgehog. The front panel of the machine is lifted as the machine drives over the cadaver, which causes the blades to stop running. In some cases, the machine withdraws and changes direction, so that only part of the dead hedgehog’s body was situated underneath the machine. The blades of the robotic lawn mower may have come into contact with the dead hedgehog but have not punctured the skin. The damages observed ranged from undetectable to the cutting of a small number of spines but might have involved minor bruising to a live hedgehog.
4	The robotic lawn mower fails to detect the presence of the hedgehog and continues to drive across it. The blades of the machine have come into contact with the dead hedgehog and have caused injuries to the cadaver. The severity of the injuries ranges from small puncture wounds on the skin (1 cm) to clipping of limbs or complete exposure of the entire abdominal region and decapitation.

**Table 3:** Coefficient estimates from the ‘position’ model.

Position	Coefficient	SE	*p*
3 + 4	−0.17	0.43	0.69
5 + 6	0.72	0.43	0.1

**Table 4:** AIC-based model selection for testing the effects of size of the hedgehog carcass on the probability of sustaining injury in collision with a robotic lawn mower.

Model	AIC	dAIC
Height sq.	162.9	0
Height	165.26	2.36
Weight	171.23	8.33
Base	171.28	8.38
Circ.	171.42	8.52
Weight sq.	172.47	9.57
Circ. sq.	173.41	10.51

**Table 5:** AIC-based model selection for testing the influence of technical features on the injury probability of the robotic lawn mowers on hedgehogs.

Model	AIC	dAIC
Drive (front vs. rear wheel)	162.28	0
Base	162.9	0.62
# wheels (3 or 4)	163.6	1.32
Wheel motor current collision detection (Y/N)	163.79	1.51
Ultrasonic sensors (Y/N)	164.18	1.9
Cutting height (mm)	164.23	1.95
Collision sensor (Y/N)	164.44	2.16
Skid plate (Y/N)	164.61	2.33
Blades (pivoting vs. fixed)	164.71	2.43
Headlights (Y/N)	164.87	2.59

## Data Availability

All relevant data from the research have been made available in the publication.

## Supplementary Materials

The following supporting information can be downloaded at: https://www.mdpi.com/article/10.3390/ani14010122/s1, Table S1: The full dataset from the tests of collisions between hedgehog carcasses and robotic lawn mowers.
