# Supplementary material for: Testing the Impact of Robotic Lawn Mowers on European Hedgehogs (Erinaceus europaeus) and Designing a Safety Test
Source: Animals (Basel). 2023 Dec 29;14(1):122. doi: 10.3390/ani14010122 (PMC10777904; doi:10.3390/ani14010122)
Supplement: Supplementary file 1 [file animals-14-00122-s001.zip › animals-2776559-supplementary.pdf]

Table S1. The full dataset from the tests of collisions between hedgehog carcasses and robotic lawn mowers.

Position	Damage Category	Year	Hedgehog	Hedgehog Weight (g)	Hedgehog Height (cm)	Hedgehog Circumference (cm)	Lawn Mower Model	Cutting Height (mm)	Blades (Pivoting P and Fixed F)	Collision Sensor (Yes/No)	Wheel Motor Current Collision Detection (Yes/No)	Wheels (3 or 4)	Front (F)-/Rear (R)-Wheel Drive	Skid Plate (Yes/No)	Headlights (Yes/No)	Ultrasonic Sensors (Yes/No)	Camera Vision (Yes/No)
1	2	2023	real	480	12	50	STIHL iMOW 7 PRO	40	P	Yes	No	4	R	No	Yes	Yes	No
2	1	2023	real	480	12	50	STIHL iMOW 7 PRO	40	P	Yes	No	4	R	No	Yes	Yes	No
3	2	2023	real	480	12	50	STIHL iMOW 7 PRO	40	P	Yes	No	4	R	No	Yes	Yes	No
4	2	2023	real	480	12	50	STIHL iMOW 7 PRO	40	P	Yes	No	4	R	No	Yes	Yes	No
5	2	2023	real	480	12	50	STIHL iMOW 7 PRO	40	P	Yes	No	4	R	No	Yes	Yes	No
6	2	2023	real	480	12	50	STIHL iMOW 7 PRO	40	P	Yes	No	4	R	No	Yes	Yes	No
1	2	2023	real	403	9	48	STIHL iMOW 5	40	P	Yes	No	4	R	No	Yes	Yes	No
2	3	2023	real	403	9	48	STIHL iMOW 5	40	P	Yes	No	4	R	No	Yes	Yes	No
3	3	2023	real	403	9	48	STIHL iMOW 5	40	P	Yes	No	4	R	No	Yes	Yes	No
4	4	2023	real	403	9	48	STIHL iMOW 5	40	P	Yes	No	4	R	No	Yes	Yes	No
5	4	2023	real	403	9	48	STIHL iMOW 5	40	P	Yes	No	4	R	No	Yes	Yes	No
6	4	2023	real	403	9	48	STIHL iMOW 5	40	P	Yes	No	4	R	No	Yes	Yes	No
1	2	2023	real (comparison)	480	12	50	STIHL iMOW 5	40	P	Yes	No	4	R	No	Yes	Yes	No
2	2	2023	real (comparison)	480	12	50	STIHL iMOW 5	40	P	Yes	No	4	R	No	Yes	Yes	No
3	2	2023	real (comparison)	480	12	50	STIHL iMOW 5	40	P	Yes	No	4	R	No	Yes	Yes	No
4	2	2023	real (comparison)	480	12	50	STIHL iMOW 5	40	P	Yes	No	4	R	No	Yes	Yes	No
5	2	2023	real (comparison)	480	12	50	STIHL iMOW 5	40	P	Yes	No	4	R	No	Yes	Yes	No
6	2	2023	real (comparison)	480	12	50	STIHL iMOW 5	40	P	Yes	No	4	R	No	Yes	Yes	No
1	4	2023	real	511	8	47	STIHL iMOW 422 P	43	F	Yes	No	4	R	No	No	No	No
2	4	2023	real	511	8	47	STIHL iMOW 422 P	43	F	Yes	No	4	R	No	No	No	No
3	3	2023	real	490	9	51	STIHL iMOW 422 P	43	F	Yes	No	4	R	No	No	No	No
4	2	2023	real	490	9	51	STIHL iMOW 422 P	43	F	Yes	No	4	R	No	No	No	No
5	3	2023	real	490	9	51	STIHL iMOW 422 P	43	F	Yes	No	4	R	No	No	No	No
6	3	2023	real	490	9	51	STIHL iMOW 422 P	43	F	Yes	No	4	R	No	No	No	No
1	4	2020	real	417			STIHL iMOW 422 P		F	Yes	No	4	R	No	No	No	No
2	4	2020	real	417			STIHL iMOW 422 P		F	Yes	No	4	R	No	No	No	No
3	4	2020	real	417			STIHL iMOW 422 P		F	Yes	No	4	R	No	No	No	No
1	3	2023	real	359	9	47	Stiga Stig-A 1500	35	P	Yes	No	4	R	No	No	No	No
2	3	2023	real	359	9	47	Stiga Stig-A 1500	35	P	Yes	No	4	R	No	No	No	No
3	1	2023	real	359	9	47	Stiga Stig-A 1500	35	P	Yes	No	4	R	No	No	No	No
4	3	2023	real	359	9	47	Stiga Stig-A 1500	35	P	Yes	No	4	R	No	No	No	No
5	4	2023	real	359	9	47	Stiga Stig-A 1500	35	P	Yes	No	4	R	No	No	No	No
6	4	2023	real	359	9	47	Stiga Stig-A 1500	35	P	Yes	No	4	R	No	No	No	No
3	2	2023	frozen	419	10.5	49	Stiga Stig-A 1500	35	P	Yes	No	4	R	No	No	No	No
1	3	2023	real	545	10	52	Segway NaviMow H3000E	67	P	Yes	No	4	R	No	No	No	No
2	3	2023	real	545	10	52	Segway NaviMow H3000E	67	P	Yes	No	4	R	No	No	No	No
3	3	2023	real	545	10	52	Segway NaviMow H3000E	67	P	Yes	No	4	R	No	No	No	No
4	4	2023	real	545	10	52	Segway NaviMow H3000E	67	P	Yes	No	4	R	No	No	No	No
5	4	2023	real	545	10	52	Segway NaviMow H3000E	67	P	Yes	No	4	R	No	No	No	No
6	3	2023	real	545	10	52	Segway NaviMow H3000E	67	P	Yes	No	4	R	No	No	No	No
3	2	2023	frozen	419	10.5	49	Segway NaviMow H3000E	67	P	Yes	No	4	R	No	No	No	No
1	1	2023	real	490	9	51	LandXScape LX812i	40	P	No	Yes	3	R	No	No	Yes	No
2	1	2023	real	490	9	51	LandXScape LX812i	40	P	No	Yes	3	R	No	No	Yes	No

3	2	2023	real	490	9	51	LandXScape LX812i	40	P	No	Yes	3	R	No	No	Yes	No
4	2	2023	real	490	9	51	LandXScape LX812i	40	P	No	Yes	3	R	No	No	Yes	No
5	2	2023	real	490	9	51	LandXScape LX812i	40	P	No	Yes	3	R	No	No	Yes	No
6	2	2023	real	490	9	51	LandXScape LX812i	40	P	No	Yes	3	R	No	No	Yes	No
1	2	2020	real	347			LandXScape LX812i		P	No	Yes	3	R	No	No	Yes	No
2	3	2020	real	347			LandXScape LX812i		P	No	Yes	3	R	No	No	Yes	No
3	4	2020	real	347			LandXScape LX812i		P	No	Yes	3	R	No	No	Yes	No
1	4	2023	real	247	7.5	40	Worx Landroid M	60	P	No	Yes	4	R	No	No	Yes	No
2	4	2023	real	241	7	38	Worx Landroid M	60	P	No	Yes	4	R	No	No	Yes	No
3	4	2023	real	237	7	39	Worx Landroid M	60	P	No	Yes	4	R	No	No	Yes	No
4	4	2023	real	237	7	39	Worx Landroid M	60	P	No	Yes	4	R	No	No	Yes	No
5	4	2023	real	237	7	39	Worx Landroid M	60	P	No	Yes	4	R	No	No	Yes	No
6	4	2023	real	237	7	39	Worx Landroid M	60	P	No	Yes	4	R	No	No	Yes	No
3	2	2023	real (comparison)	375	9	46	Worx Landroid M	60	P	No	Yes	4	R	No	No	Yes	No
4	3	2023	real (comparison)	375	9	46	Worx Landroid M	60	P	No	Yes	4	R	No	No	Yes	No
5	3	2023	real (comparison)	375	9	46	Worx Landroid M	60	P	No	Yes	4	R	No	No	Yes	No
6	4	2023	real (comparison)	375	9	46	Worx Landroid M	60	P	No	Yes	4	R	No	No	Yes	No
1	4	2023	real (comparison)	375	9	46	Worx Landroid M	60	P	No	Yes	4	R	No	No	Yes	No
2	4	2023	real (comparison)	375	9	46	Worx Landroid M	60	P	No	Yes	4	R	No	No	Yes	No
3	2	2023	frozen	419	10.5	49	Worx Landroid M	60	P	No	Yes	4	R	No	No	Yes	No
1	3	2020	real	422			Worx Landroid M		P	No	Yes	4	R	No	No	Yes	No
2	3	2020	real	422			Worx Landroid M		P	No	Yes	4	R	No	No	Yes	No
3	2	2020	real	422			Worx Landroid M		P	No	Yes	4	R	No	No	Yes	No
1	3	2023	real	482	9	47	Worx Landroid L	60	P	No	Yes	4	R	No	No	No	No
2	4	2023	real	482	9	47	Worx Landroid L	60	P	No	Yes	4	R	No	No	No	No
3	2	2023	real	482	9	47	Worx Landroid L	60	P	No	Yes	4	R	No	No	No	No
4	2	2023	real	482	9	47	Worx Landroid L	60	P	No	Yes	4	R	No	No	No	No
5	3	2023	real	482	9	47	Worx Landroid L	60	P	No	Yes	4	R	No	No	No	No
6	4	2023	real	482	9	47	Worx Landroid L	60	P	No	Yes	4	R	No	No	No	No
1	4	2020	real	422			Worx Landroid L		P	No	Yes	4	R	No	No	No	No
2	3	2020	real	422			Worx Landroid L		P	No	Yes	4	R	No	No	No	No
3	3	2020	real	422			Worx Landroid L		P	No	Yes	4	R	No	No	No	No
1	2	2023	real	490	9	51	Kress Mission KR111	45	P	Yes	No	4	R	No	No	Yes	No
2	2	2023	real	490	9	51	Kress Mission KR111	45	P	Yes	No	4	R	No	No	Yes	No
3	4	2023	real	490	9	51	Kress Mission KR111	45	P	Yes	No	4	R	No	No	Yes	No
4	2	2023	real	490	9	51	Kress Mission KR111	45	P	Yes	No	4	R	No	No	Yes	No
5	4	2023	real	490	9	51	Kress Mission KR111	45	P	Yes	No	4	R	No	No	Yes	No
6	2	2023	real	490	9	51	Kress Mission KR111	45	P	Yes	No	4	R	No	No	Yes	No
1	4	2020	real	344			Kress Mission KR111		P	Yes	No	4	R	No	No	Yes	No
2	4	2020	real	344			Kress Mission KR111		P	Yes	No	4	R	No	No	Yes	No
3	4	2020	real	344			Kress Mission KR111		P	Yes	No	4	R	No	No	Yes	No
1	4	2023	real	290	7.5	40	Husqvarna Nera	43	P	Yes	No	4	R	Yes	Yes	Yes	No
2	4	2023	real	290	7.5	40	Husqvarna Nera	43	P	Yes	No	4	R	Yes	Yes	Yes	No
3	4	2023	real	318	9	40	Husqvarna Nera	43	P	Yes	No	4	R	Yes	Yes	Yes	No
4	2	2023	real	318	9	40	Husqvarna Nera	43	P	Yes	No	4	R	Yes	Yes	Yes	No
5	2	2023	real	318	9	40	Husqvarna Nera	43	P	Yes	No	4	R	Yes	Yes	Yes	No
6	2	2023	real	318	9	40	Husqvarna Nera	43	P	Yes	No	4	R	Yes	Yes	Yes	No
3	0	2023	real (comparison)	419	9	57	Husqvarna Nera	43	P	Yes	No	4	R	Yes	Yes	Yes	No
3	3	2023	real (comparison)	419	9	57	Husqvarna Nera	43	P	Yes	No	4	R	Yes	Yes	Yes	No
3	2	2023	frozen	192	8	37	Husqvarna Nera	43	P	Yes	No	4	R	Yes	Yes	Yes	No
1	1	2023	real (comparison)	419	9	57	Husqvarna Aspire R4	50	P	No	Yes	3	F	No	No	No	No
2	1	2023	real (comparison)	419	9	57	Husqvarna Aspire R4	50	P	No	Yes	3	F	No	No	No	No

3	1	2023	real (comparison)	419	9	57	Husqvarna Aspire R4	50	P	No	Yes	3	F	No	No	No	No
4	2	2023	real (comparison)	419	9	57	Husqvarna Aspire R4	50	P	No	Yes	3	F	No	No	No	No
5	1	2023	real (comparison)	419	9	57	Husqvarna Aspire R4	50	P	No	Yes	3	F	No	No	No	No
6	1	2023	real (comparison)	419	9	57	Husqvarna Aspire R4	50	P	No	Yes	3	F	No	No	No	No
3	2	2023	frozen	192	8	37	Husqvarna Aspire R4	50	P	No	Yes	3	F	No	No	No	No
1	1	2023	real	246	6.5	38	Husqvarna Aspire R4	50	P	No	Yes	3	F	No	No	No	No
2	1	2023	real	246	6.5	38	Husqvarna Aspire R4	50	P	No	Yes	3	F	No	No	No	No
3	1	2023	real	246	6.5	38	Husqvarna Aspire R4	50	P	No	Yes	3	F	No	No	No	No
4	2	2023	real	246	6.5	38	Husqvarna Aspire R4	50	P	No	Yes	3	F	No	No	No	No
5	1	2023	real	246	6.5	38	Husqvarna Aspire R4	50	P	No	Yes	3	F	No	No	No	No
6	4	2023	real	246	6.5	38	Husqvarna Aspire R4	50	P	No	Yes	3	F	No	No	No	No
1	1	2023	real	318	9	40	Husqvarna Automower 450X	60	P	Yes	No	4	R	Yes	Yes	Yes	No
2	2	2023	real	318	9	40	Husqvarna Automower 450X	60	P	Yes	No	4	R	Yes	Yes	Yes	No
3	1	2023	real	318	9	40	Husqvarna Automower 450X	60	P	Yes	No	4	R	Yes	Yes	Yes	No
4	2	2023	real	318	9	40	Husqvarna Automower 450X	60	P	Yes	No	4	R	Yes	Yes	Yes	No
5	1	2023	real	318	9	40	Husqvarna Automower 450X	60	P	Yes	No	4	R	Yes	Yes	Yes	No
6	1	2023	real	318	9	40	Husqvarna Automower 450X	60	P	Yes	No	4	R	Yes	Yes	Yes	No
1	1	2020	real	422			Husqvarna Automower 450X		P	Yes	No	4	R	Yes	Yes	Yes	No
2	2	2020	real	422			Husqvarna Automower 450X		P	Yes	No	4	R	Yes	Yes	Yes	No
3	2	2020	real	422			Husqvarna Automower 450X		P	Yes	No	4	R	Yes	Yes	Yes	No
3	1	2023	real (comparison)	419	9	57	Husqvarna Automower 450X	60	P	Yes	No	4	R	Yes	Yes	Yes	No
3	2	2023	frozen	192	8	37	Husqvarna Automower 450X	60	P	Yes	No	4	R	Yes	Yes	Yes	No
1	4	2023	real	237	7	39	Husqvarna Automower 310	65	P	Yes	No	4	R	Yes	No	No	No
2	3	2023	real	237	7	39	Husqvarna Automower 310	65	P	Yes	No	4	R	Yes	No	No	No
3	3	2023	real	237	7	39	Husqvarna Automower 310	65	P	Yes	No	4	R	Yes	No	No	No
4	3	2023	real	237	7	39	Husqvarna Automower 310	65	P	Yes	No	4	R	Yes	No	No	No
5	3	2023	real	237	7	39	Husqvarna Automower 310	65	P	Yes	No	4	R	Yes	No	No	No
6	3	2023	real	237	7	39	Husqvarna Automower 310	65	P	Yes	No	4	R	Yes	No	No	No
3	1	2023	real (comparison)	419	9	57	Husqvarna Automower 310	65	P	Yes	No	4	R	Yes	No	No	No
3	2	2023	frozen	192	8	37	Husqvarna Automower 310	65	P	Yes	No	4	R	Yes	No	No	No
1	2	2023	real	318	9	40	Husqvarna Automower 305	52	P	No	Yes	4	R	Yes	No	No	No
2	1	2023	real	318	9	40	Husqvarna Automower 305	52	P	No	Yes	4	R	Yes	No	No	No
3	2	2023	real	318	9	40	Husqvarna Automower 305	52	P	No	Yes	4	R	Yes	No	No	No
4	2	2023	real	318	9	40	Husqvarna Automower 305	52	P	No	Yes	4	R	Yes	No	No	No
5	1	2023	real	318	9	40	Husqvarna Automower 305	52	P	No	Yes	4	R	Yes	No	No	No
6	1	2023	real	318	9	40	Husqvarna Automower 305	52	P	No	Yes	4	R	Yes	No	No	No
1	2	2020	real	422			Husqvarna Automower 305		P	No	Yes	4	R	Yes	No	No	No
2	2	2020	real	422			Husqvarna Automower 305		P	No	Yes	4	R	Yes	No	No	No
3	2	2020	real	422			Husqvarna Automower 305		P	No	Yes	4	R	Yes	No	No	No
3	2	2023	real (comparison)	419	9	57	Husqvarna Automower 305	52	P	No	Yes	4	R	Yes	No	No	No
5	4	2023	real (comparison)	419	9	57	Husqvarna Automower 305	52	P	No	Yes	4	R	Yes	No	No	No
6	4	2023	real (comparison)	419	9	57	Husqvarna Automower 305	52	P	No	Yes	4	R	Yes	No	No	No
5	2	2023	real (comparison)	419	9	57	Husqvarna Automower 305	52	P	No	Yes	4	R	Yes	No	No	No
6	4	2023	real (comparison)	419	9	57	Husqvarna Automower 305	52	P	No	Yes	4	R	Yes	No	No	No
3	2	2023	frozen	192	8	37	Husqvarna Automower 305	52	P	No	Yes	4	R	Yes	No	No	No
1	3	2023	real	241	7	38	Husqvarna Automower 105	45	P	Yes	No	3	F	Yes	No	No	No
2	3	2023	real	241	7	38	Husqvarna Automower 105	45	P	Yes	No	3	F	Yes	No	No	No
3	4	2023	real	241	7	38	Husqvarna Automower 105	45	P	Yes	No	3	F	Yes	No	No	No
4	4	2023	real	241	7	38	Husqvarna Automower 105	45	P	Yes	No	3	F	Yes	No	No	No
5	4	2023	real	241	7	38	Husqvarna Automower 105	45	P	Yes	No	3	F	Yes	No	No	No
6	4	2023	real	241	7	38	Husqvarna Automower 105	45	P	Yes	No	3	F	Yes	No	No	No
1	2	2020	real	422			Husqvarna Automower 105		P	Yes	No	3	F	Yes	No	No	No

2	2	2020	real	422			Husqvarna Automower 105		P	Yes	No	3	F	Yes	No	No	No
3	1	2020	real	422			Husqvarna Automower 105		P	Yes	No	3	F	Yes	No	No	No
3	2	2023	real (comparison)	419	9	57	Husqvarna Automower 105	45	P	Yes	No	3	F	Yes	No	No	No
5	3	2023	real (comparison)	419	9	57	Husqvarna Automower 105	45	P	Yes	No	3	F	Yes	No	No	No
6	4	2023	real (comparison)	419	9	57	Husqvarna Automower 105	45	P	Yes	No	3	F	Yes	No	No	No
3	2	2023	frozen	192	8	37	Husqvarna Automower 105	45	P	Yes	No	3	F	Yes	No	No	No
1	3	2023	real	229	5.5	38	Honda Miimo HRM 40 Live	47	P	Yes	Yes	4	R	No	No	No	No
2	3	2023	real	229	5.5	38	Honda Miimo HRM 40 Live	47	P	Yes	Yes	4	R	No	No	No	No
3	3	2023	real	229	5.5	38	Honda Miimo HRM 40 Live	47	P	Yes	Yes	4	R	No	No	No	No
4	4	2023	real	229	5.5	38	Honda Miimo HRM 40 Live	47	P	Yes	Yes	4	R	No	No	No	No
5	3	2023	real	229	5.5	38	Honda Miimo HRM 40 Live	47	P	Yes	Yes	4	R	No	No	No	No
6	3	2023	real	229	5.5	38	Honda Miimo HRM 40 Live	47	P	Yes	Yes	4	R	No	No	No	No
1	1	2020	real	515			Honda Miimo HRM 40 Live		P	Yes	Yes	4	R	No	No	No	No
2	3	2020	real	515			Honda Miimo HRM 40 Live		P	Yes	Yes	4	R	No	No	No	No
3	1	2020	real	515			Honda Miimo HRM 40 Live		P	Yes	Yes	4	R	No	No	No	No
3	3	2023	real (comparison)	419	9	57	Honda Miimo HRM 40 Live	47	P	Yes	Yes	4	R	No	No	No	No
3	4	2023	frozen	192	8	37	Honda Miimo HRM 40 Live	47	P	Yes	Yes	4	R	No	No	No	No
1	3	2023	real	247	7.5	40	Gardena Sileno Life	35	P	No	Yes	4	F	No	No	No	No
2	1	2023	real	247	7.5	40	Gardena Sileno Life	35	P	No	Yes	4	F	No	No	No	No
3	1	2023	real	247	7.5	40	Gardena Sileno Life	35	P	No	Yes	4	F	No	No	No	No
4	1	2023	real	247	7.5	40	Gardena Sileno Life	35	P	No	Yes	4	F	No	No	No	No
5	3	2023	real	247	7.5	40	Gardena Sileno Life	35	P	No	Yes	4	F	No	No	No	No
6	3	2023	real	247	7.5	40	Gardena Sileno Life	35	P	No	Yes	4	F	No	No	No	No
1	2	2020	real	422			Gardena Sileno Life		P	No	Yes	4	F	No	No	No	No
2	2	2020	real	422			Gardena Sileno Life		P	No	Yes	4	F	No	No	No	No
3	1	2020	real	422			Gardena Sileno Life		P	No	Yes	4	F	No	No	No	No
3	1	2023	real (comparison)	419	9	57	Gardena Sileno Life	35	P	No	Yes	4	F	No	No	No	No
3	1	2023	frozen	192	8	37	Gardena Sileno Life	35	P	No	Yes	4	F	No	No	No	No
1	3	2023	real	229	5.5	38	Gardena Sileno City	58	P	No	Yes	3	F	No	No	No	No
2	3	2023	real	229	5.5	38	Gardena Sileno City	58	P	No	Yes	3	F	No	No	No	No
3	3	2023	real	229	5.5	38	Gardena Sileno City	58	P	No	Yes	3	F	No	No	No	No
4	4	2023	real	229	5.5	38	Gardena Sileno City	58	P	No	Yes	3	F	No	No	No	No
5	4	2023	real	229	5.5	38	Gardena Sileno City	58	P	No	Yes	3	F	No	No	No	No
6	3	2023	real	229	5.5	38	Gardena Sileno City	58	P	No	Yes	3	F	No	No	No	No
1	3	2020	real	422			Gardena Sileno City		P	No	Yes	3	F	No	No	No	No
2	3	2020	real	422			Gardena Sileno City		P	No	Yes	3	F	No	No	No	No
3	3	2020	real	422			Gardena Sileno City		P	No	Yes	3	F	No	No	No	No
3	2	2023	real (comparison)	419	9	57	Gardena Sileno City	58	P	No	Yes	3	F	No	No	No	No
3	2	2023	frozen	192	8	37	Gardena Sileno City	58	P	No	Yes	3	F	No	No	No	No
1	4	2023	real	375	9	46	AL-KO Robolinho 1150 W	50	F	No	Yes	4	R	No	No	No	No
2	3	2023	real	375	9	46	AL-KO Robolinho 1150 W	50	F	No	Yes	4	R	No	No	No	No
3	3	2023	real	375	9	46	AL-KO Robolinho 1150 W	50	F	No	Yes	4	R	No	No	No	No
4	3	2023	real	375	9	46	AL-KO Robolinho 1150 W	50	F	No	Yes	4	R	No	No	No	No
5	4	2023	real	375	9	46	AL-KO Robolinho 1150 W	50	F	No	Yes	4	R	No	No	No	No
6	3	2023	real	375	9	46	AL-KO Robolinho 1150 W	50	F	No	Yes	4	R	No	No	No	No
1	4	2020	real	377			AL-KO Robolinho 1150 W		F	No	Yes	4	R	No	No	No	No
2	3	2020	real	377			AL-KO Robolinho 1150 W		F	No	Yes	4	R	No	No	No	No
3	4	2020	real	377			AL-KO Robolinho 1150 W		F	No	Yes	4	R	No	No	No	No
